# Kinetic stability of all-in-one parenteral nutrition admixtures in the presence of high dose Ca^2+^ additive under clinical application circumstances

**DOI:** 10.1186/1475-2891-11-32

**Published:** 2012-05-16

**Authors:** István G Télessy, Judith Balogh, Barnabás Szabó, Ferenc Csempesz, Romána Zelkó

**Affiliations:** 1 MedBioFit Research & Organization L.p.c, Fácán Street 25, 2100, Gödöllö, Hungary; 2 University Pharmacy Department of Pharmacy Administration, Semmelweis University, 1092, Budapest, Hőgyes E. Street 7-9, Hungary; 3 Department of Colloid Chemistry and Technology, Eötvös L. University, Pázmány Péter Avenue 1/C, 1117, Budapest, Hungary

**Keywords:** Kinetic stability, All-in-one parenteral nutrition admixture, Droplet size distribution

## Abstract

**Background:**

TPN infusions are usually administered during a treatment period of 10–24 hours per day due to the metabolic capacity of the liver. During this time interval physicochemically stable TPN solution (emulsion) is needed for the treatment. The purpose of the present study was to examine how the kinetic stability features of ready-made total parenteral nutrition admixtures containing olive oil and soybean oil will change under the usage-modeling 24-hour application with and without overdose Ca^2+^.

**Methods:**

Particle size analysis and zeta potential measurements were carried out to evaluate the possible changes in the kinetic stability of the emulsions.

**Results:**

Our results indicate that in two of the four mixtures bimodal droplet-size distribution figures were detected and appearance of fat particles over 5 μm can not be disclosed. The tendency for separation of large diameter droplets in the two types of oil-based emulsion systems was different. In case of soybean containing emulsion second peak of droplets appeared in the bottom of the container in contrast to the olive oil containing emulsions where the second peak appeared in the surface layer. Interestingly this phenomenon is independent of calcium-content.

**Conclusions:**

From therapeutic point the emulsions of the bigger droplets containing upper layer are safer because the potentially dangerous big droplets could remain in the infusion bag after the administration.

## Background

### Total parenteral nutrition admixtures

Since over two decades it is well-known that good availability of nutrients can be reached by simultaneous administration of macronutrients. Clinical nutrition therefore is made by use of nutrient admixtures. Should we concentrate to the intravenous nutrition, these solutions are basically composed of amino acids, glucose and fat emulsions therefore they are called as all-in-one mixtures. Usually, additional important ingredients like electrolytes, trace elements and vitamins, are also concomitantly admixed and the final product is named as total parenteral nutrient (TPN) mixture [1]. There are special conditions (e.g. high risk of tetanic contraction or electrolyte depletion in case of cardiac surgery with extracorporeal circulation) when extra electrolytes are needed. Typically Ca^2+^ and phosphate are the boosted ions out of which Ca^2+^ is more dangerous in oil-containing mixtures due to the bivalent cation property that can damage lipid-droplet surface charge and so the repulsive force. TPN infusions are usually administered during a treatment period of 10–24 hours per day due to the metabolic capacity of the liver.

During this time interval physicochemically stable TPN solution (emulsion) is needed for the treatment. In our previous studies the droplet size stability of all-in-one nutritive mixtures containing different fat emulsions were studied. Authors concluded that droplet stability of MCT/LCT lipids surpassed the pure LCT [2] and the SMOF-lipid was more stable than that of MCT/LCT [3]. In our present study we broadened the range of lipids with olive-oil containing emulsion. The olive oil consists of mainly MUFA that has a lot of beneficial properties, primarily the stability against lipid-peroxidation [4]. This is important in case of storage because in the plastic (EVA) bags used for storage of TPNs peroxidation of lipids is much more pronounced than that in glass bottles [5]. In human studies, however the protective action against oxidative stress is controversial [6,7]. Aging process in oil-containing colloid systems may increase critical flocculation concentration of eg. Ca^2+^ in the emulsions [8] and the lipid-peroxidation can play a role in the aging processes of colloid systems like oil-containing TPNs, this property of olive oil can be of importance in case of kinetic/physical stability of all-in-one nutrient mixtures however, we did not study the details of aging process itself in this experiment.

The aim of the present study was to demonstrate any clinically significant physicochemical difference between two TPNs containing LCT-based lipid emulsion regarding the droplet-size and zeta potential.

### Physicochemical considerations

Total parenteral nutrition admixtures are colloid dispersion systems, where droplet-size should be under 5 μm in long-term. This size limit remains under the human red blood cell diameter (6–8 μm) and so producers and users can avoid occlusion of capillaries due to the fat-droplets in the infusion. Stable lipid-emulsions used in the iv therapy are usually monodisperse systems with a mean droplet diameter of 200–600 nm, and according to the Gauss-distribution with lower and upper limits of 95% at ca. 50 and 1000 nm respectively. According to the USP droplets with diameter higher than 5 μm are still allowed in parenteral lipid emulsions and in therapeutic admixtures but they must not exceed the proportion of 0.05% [9]. In emulsions used as human pharmaceuticals the droplets are usually stabilized with any type of lecithin. The Stern-layer around the small fat globules in the water-based emulsions like all-in-one iv nutritions, is a stable interface where the surrounding ions are strongly bound to the globules. The stability of this system depends on the repulsive and attractive colloidal forces, such as van der Waals, electrical double-layer and steric forces that act between the droplets. The repulsive force on the surface of the fat droplet can be measured as zeta-potential. In case of this type of emulsions negative charge of oil droplets below −30 mV means that the system is stable enough to be used for intravenous nourishment of patients [10]. Should we affect the density of the Stern-layer around the droplets by altering the conductivity (eg. addition of bivalent cations) we jeopardize the stability of the whole system, therefore monitoring of zeta-potential and the droplet-size in a new composition is essential before using it for treatment of patients.

## Methods

### Materials

Industrial convenience systems of OliClinomel N6-900 E (Baxter) and Kabiven (FreseniusKabi) were used as all-in-one nutrient mixtures. OliClinomel contains 80% of olive oil (LCT, in 60-80% esterified by C18-MUFA of the ω-9 subgroup) and 20% of soybean oil (LCT, mainly triglyceride esters composed of C-18:2 PUFA of the ω-6 subgroup). Kabiven contains 100% soybean oil (LCT, mainly C18:2 PUFA esters). The three-chamber bags were reconstituted according to the instruction for use by breaking the non-permanent seals between the compartments and then mixing them by inverting the bag [10].

An overdose of Ca-gluconate (25 ml into 1000 ml out of 100 mg/ml injection = 5.5 mmol/l Ca^2+^ extra) was added during the preparation process to one of the TPN. Ingredients in g/l and mmol/l after reconstitution and additional Ca-gluconate are as follows:

OliClinomel (samples OA and OC) Macronutrients: 34 g/l Amino acids, 120 g/l Glucose, 40 g/l FatMicronutrient: 32 mmol/l Na^+^, 24 mmol/l K^+^, 2.2 mmol/l Mg^2+^, 2 mmol/l (Sample OA) and 7.5 mmol/l (Sample OC) Ca^2+^, 10 mmol/l Phosphate, 53 mmol/l Acetate and 46 mmol/l Cl^-^.

Kabiven (samples KA and KC) Macronutrients: 33 g/l Amino acids, 100 g/l Glucose, 39 g/l FatMicronutrients: 31 mmol/l Na^+^, 23 mmol/l K^+^, 3.9 mmol/l Mg^2+^, 1.9 mmol/l (Sample KA) and 7.4 mmol/l (Sample KC) Ca^2+^, 9.7 mmol/l Phosphate, 3.9 mmol/l Sulphate, 38 mmol/l Acetate and 45 mmol/l Cl^-^.

Previously it was found that a moderate difference (up to 20%) in glucose content did not influence the droplet-stability under very similar electrolyte-environment [3]. So we can state that the two TPN systems studied in our present experiments are comparable. Test mixtures signed with C contain ca. 50% more Ca^2+^ than maximally advised by the manufacturers and it was considered the possible upper limit of Ca^2+^ content in the comparison of the stability of different mixtures.

### Sampling

After shaking the solution container, it has been connected to an infusion set and hanged up to an infusion stander. Light protection was not used and the storage temperature was hospital ward room-temperature (22–24°C). After a lag time of 5 minutes infusion administration with a speed of 2 ml/min (= 120 ml/hours) has been started. Samples for droplet-size control measurements were taken from the “supernatant” of the infusion bag content (within 3 mm of the surface, later mentioned as “upper level sample” signed as “up”) and from the infusion tube 25 cm after the output port of the container bag (“lower level sample” in short “low”). Samples (5 ml) have been taken just after the start (zero point) and 24 hours later (end point). For zeta-potential measurements we took samples from the middle of the container and sampling was done at start, after 1, 2, 3, 10, 11, 12 hours to simulate the real parenteral nutrition conditions and in the end of the study period (24. hour).

### Laboratory measurements

Droplet size measurements were performed by photon-correlation spectroscopy (PCS, made by Zetasizer 4 and Nanosizer ZS, Malvern Instruments, England) and the surface-charge was checked by measurement of electrophoretic movement (Laser Doppler Anemometry by Zetasizer 4, Malvern Instruments, England). Droplet size distributions and zeta-potential figures were made out of 2 or 3 separate samples in each measurement depending on the coherence of measurement results. Nature samples were diluted by purified water in a proportion of 1:300 in order to reach the measurement concentration-range according to the manual for use of the equipment. The measurement limits of the Zetasizer 4 equipment are 3-3000 nm for determination of droplet size and 20-20000 nm in case of LDA [11]. The measuring range of the Zetasizer Nano ZS equipment (Dynamic Light Scattering principle) for particle size measurement is between 0.3 nm-10 μm and for zeta-potential 3.8-100000 nm [12]. We expected the 99.5% of droplets in the range of 10-3000 nm with the main peak between 300-700 nm and the critical point was the size over 5 μm. Surface charge of droplets was expected between −40 and −10 mV. Droplet-size control was performed according to the accepted rules based on the USP [13,14]. We also controlled the droplet stability by electrokinetic (zeta-potential) measurement as advised [15].

## Results and discussion

**Figure 1:**
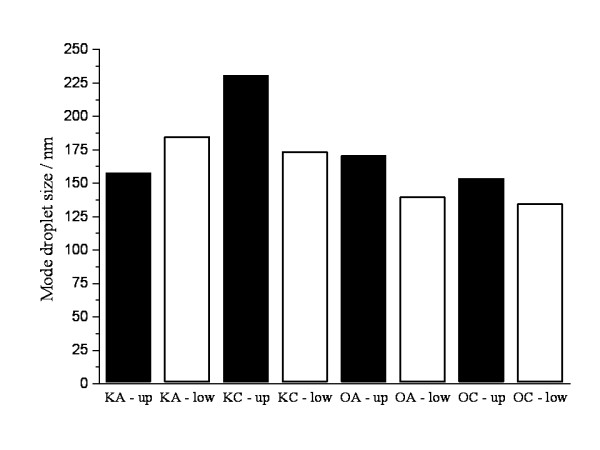
Mode droplet size values of different fractions of admixtures at the initial (without up or low index) point and after 24 hours.

In our study we simulated the two endpoints of a typical clinical situation of nutrition therapy. Therefore the duration of inspection was just 24 hours. Under clinical conditions one dose of TPN (usually 1–3 litres for adults) is not administered in a shorter period than 8 hours and not longer than 24 hours. After preparation of the iv solutions we immediately checked the main parameters according to the above mentioned schedule. The comparison of the mode droplet-size values shows that different nutrient test-solutions with and without additional Ca^2+^ are within the expected droplet size range of 0.2-0.6 μm. The MDS situation of different test mixtures at beginning and at the end of the 24 hour of infusion did not change significantly (Figure 1). The large-size tail of the distribution-figure was analyzed at the end of the study in both sites of surface layer and after the port. At 24 hours we realized that in the supernatant phase and in the infusion line small proportion of droplets over 5 μm could be detected. Intensity-weighted distributions (Table 1) demonstrate that only in infusion line samples of Kabimix and in the supernatant phase of OliClinomel appeared a second peak of 2552-2883 nm (KA) and 1851-3006 nm (KC) as well as 2277-2816 nm (OA) and 2265-2895 nm (OC) respectively. Exact proportion of over 5 μm droplets could not be estimated in this study.

**Table 1:** Appearance of second peak in droplet size and their ratios

**Sample**	**Droplet size and intensity ratio**			
	**1st peak (nm)**	**Intensity ratio**	**2nd peak (nm)**	**Intensity ratio**
KA-up	158	100	0	0
KA-low	185	97.5	2545	2.5
KC-up	231	100	0	0
KC-low	174	93.3	2290	6.7
OA-up	171	95.2	2115	4.8
OA-low	1401	100	0	0
OC-up	154	96	2343	4
OC-low	135	100	0	0

Electrokinetic properties of emulsion were demonstrated by measurement of zeta-potential. Test-results explain that mean charge of droplet surface in all samples remained under −30 mV (Table 2). It means that the electrokinetic parameter supports droplet size stability features measured with globule-size distribution techniques.

**Table 2:** Mean surface charge (mV) of droplets in different TPN mixtures in course of the nutrition period

**Sample**	**Nutrition period (h)**							
	**0**	**1**	**2**	**3**	**10**	**11**	**12**	**24**
	**Mean surface charge (mV)**							
KA	−39.10	−37.80	−38.30	−38.00	−38.10	−37.60	−36.20	−36.20
KC	−34.00	−33.50	−34.60	−33.50	−33.40	−32.60	−38.30	−38.30
OA	−40.00	−40.10	−42.00	−41.10	−40.90	−41.00	−40.40	−40.40
OC	−36.40	−35.70	−35.60	−35.70	−35.90	−35.80	−35.40	−35.40

The ageing of emulsions usually brings increase in droplet-size. In clinical use droplet size stability should be reserved during the time of infusion, in case of intravenous nutrition within 24 hours as well. Our results demonstrate that vast majority of droplet remained within the safe range of under 5000 nm and zeta-potential remained under the safe value of −30 mV. However, in some mixtures oversize droplets were also detected and according to the USP rules fat droplets over 5 μm must not exceed the proportion of 0.05%. Proportions of second peaks are small (Table 1), but this range of globule-size is on the borderline and exact number of fat particles over 5000 nm has not been estimated in this experiment. Therefore, from security reasons in these cases use of lipid-filter is reasonable.

It was found an interesting phenomenon. The tendency for separation of large diameter droplets in the two emulsion systems is different. In case of Kabiven second peak of droplets appears in the bottom of the container in contrast to OliClinomel where the second peak appears in the samples taken from the surface layer. Interestingly this phenomenon is independent of calcium-content. From therapeutic point of view we can state that emulsions where the bigger droplets are in the upper layer are safer because infusions are usually infused with a small residual volume and the potentially dangerous big droplets remain in the infusion bag. The difference between the two types of TPN mixtures could be partly explained by the different glucose content and its effect on the osmolarity and the slightly different density of the two emulsions. The higher osmolarity (1190 mOsm/l vs 1060 mOsm/l) and lower density (1.039 g/ml vs 1.049 g/ml) of OliClinomel resulted in droplets of higher particle sizes in the therapeutically safer surface layer [16]. Further targeted studies can elucidate this phenomenon. Maybe the difference in composition of the oversize droplets could explain the experienced deviation. Nevertheless it is unlikely that droplet-size differences are results of olive oil or soybean oil content.

## Conclusion

Zeta-potential of the droplets in experimental emulsions proved the stability of the colloid system, but size-distribution showed disparity within the test mixtures. In two of the four mixtures bimodal droplet-size distribution figures were detected and appearance of fat particles over 5 μm can not be disclosed. The bimodality was independent of the Ca^2+^ content. Until the destabilizing component is not identified and the consequent different aggregation behavior of the two types of TPN is not clarified, the lipid filter usage is advised in the course of the parenteral nutrition.

## Abbreviations

TPN: Total parenteral nutrition; MCT: Medium chain triglyceride; LCT: Long chain triglyceride; SMOF: Soybean oil, medium chain triglyceride, olive oil and fish oil; MUFA: Mono-unsaturated fatty acid; USP: United States pharmacopoeia; MDS: Mode droplet size; iv: Intravenous.

## Competing interests

The authors declare that they have no competing interests.

## Authors’ contributions

I.G. Télessy participated in the design of the study and the manuscript. B. Szabó carried out the droplet size measurements and helped to draft the manuscript. B. Judit participated in the design of the study and the preparation of the experimental setup. F. Csempesz carried out the zeta potential measurements and critically evaluated the results. R. Zelkó conceived of the study, and participated in its design and coordination and helped to draft the manuscript. All authors read and approved the final manuscript.
